# Prey preferences of the chimpanzee (*Pan troglodytes*)

**DOI:** 10.1002/ece3.7633

**Published:** 2021-05-04

**Authors:** Cassandra K. Bugir, Thomas M. Butynski, Matt W. Hayward

**Affiliations:** ^1^ Conservation Biology Research Group School of Environmental and Life Sciences University of Newcastle Callaghan NSW Australia; ^2^ Eastern Africa Primate Diversity and Conservation Program Lolldaiga Hills Research Programme Nanyuki Kenya; ^3^ Mammal Research Institute University of Pretoria Tshwane South Africa; ^4^ Centre for African Conservation Ecology Nelson Mandela University Port Elizabeth South Africa

**Keywords:** chimpanzee, hunter‐gatherer, hunting, Jacobs’ index, optimal foraging theory, prey preference, sex ratio

## Abstract

The common chimpanzee *Pan troglodytes* is the closest extant relative of modern humans and is often used as a model organism to help understand prehistoric human behavior and ecology. Originally presumed herbivorous, chimpanzees have been observed hunting 24 species of birds, ungulates, rodents, and other primates, using an array of techniques from tools to group cooperation. Using the literature on chimpanzee hunting behavior and diet from 13 studies, we aimed to determine the prey preferences of chimpanzees. We extracted data on prey‐specific variables such as targeted species, their body weight, and their abundance within the prey community, and hunter‐specific variables such as hunting method, and chimpanzee group size and sex ratio. We used these data in a generalized linear model to determine what factors drive chimpanzee prey preference. We calculated a Jacobs’ index value for each prey species killed at two sites in Uganda and two sites in Tanzania. Chimpanzees prefer prey with a body weight of 7.6 ± 0.4 kg or less, which corresponds to animals such as juvenile bushbuck (*Tragelaphus scriptus*) and adult ashy red colobus monkeys (*Piliocolobus tephrosceles*). Sex ratio in chimpanzee groups is a main driver in developing these preferences, where chimpanzees increasingly prefer prey when in proportionally male‐dominated groups. Prey preference information from chimpanzee research can assist conservation management programs by identifying key prey species to manage, as well as contribute to a better understanding of the evolution of human hunting behavior.

## Graphical abstract

Using published literature on chimpanzee dietary behavior, we were able to extract information to determine their preferred prey.

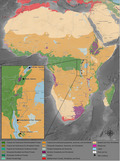

## 1 INTRODUCTION

Modern humans (*Homo sapiens*) share 96% of their genome with chimpanzees (*Pan* *troglodytes*; Tomkins, 2016). This shared ancestry means that chimpanzees are often used as a model for understanding early hominid behavioral ecology (Pilbeam et al., 2017). Originally, chimpanzees were presumed to be herbivorous (Stanford, 1995). It is now known, however, that they actively hunt animals, including primates, small ungulates, birds, reptiles, and invertebrates, which account for up to 4% of the diet (Boesch, 2002; Gilby & Wawrzyniak, 2018; Mitani & Watts, 2001). Prey is highly sought after, sometimes with the use of tools and group hunting parties (Stanford et al., 1994). Chimpanzee hunting behaviors are particularly relevant in determining the role that predation played in hominid evolution, as well as in the evolution of group hunting strategies (Pruetz et al., 2015; Stanford, 1996).

The evolution of chimpanzee prey preferences can be explained by the optimal foraging theory, which predicts prey will be selected based on a cost/benefit relationship between the energetic benefits of consuming a prey item compared with the costs of capturing and ingesting it without getting injured (Pyke, 1984). Chimpanzee hunting and the acquisition of preferential prey vary from group to group, forming group cultural identities. In particular, chimpanzees in home ranges that have heavy seasonal differences that affect food availability, prefer high quality (i.e., more energy maximizing) prey and avoid low quality prey (Newton‐Fisher et al., 2007). Under the optimality theory paradigm, we predict that chimpanzees prefer the largest prey that can be safely captured and killed. Depending on the cultural traditions within the group, transfer of meat or access to carcasses goes to mature members within the group (Hohmann et al., 2009). This behavior encourages collaboration among hunters and provides an energetic benefit (Goodall, 1986), thereby minimizing cost relative to solitary hunting.

Using the published literature on chimpanzee hunting behavior and diet, we aimed to determine the prey preferences of chimpanzees and the factors that contribute to preferential prey acquisition.

## 2 METHODS

To assess the prey selection of chimpanzees, we followed the methods of Hayward et al. (2006, 2017). We conducted a primary literature search using JSTOR, Science Direct, Elsevier, and Google Scholar for the following keywords: “chimpanzee” or “Pan troglodytes” AND “prey preference” OR “hunt*” OR “diet” OR “predation” OR “hunting strategies” OR “food shar*” OR “meat shar*”. Studies that did not have sufficient data were excluded from consideration. Studies where only one or two prey species were listed, or those that only provided qualitative data, were considered to have insufficient. Where only kill or abundance data were provided, the authors were contacted for supplementary information, or we contacted other authors who worked at the same site at about the same time (±5 years). If an author did not respond, we searched for abundance information for the same study area around the same year using Google Scholar.

The crucial information needed for this study from each site included prey species and their absolute or relative population abundance or density, number of kills, hunting method (solitary or group/hunting parties), hunting group size, sex ratio of group, and prey body weight in kilograms (kg). The methods by which chimpanzees hunt prey are typically recorded in each publication, as groups use different methods—whether through solitary hunting, hunting parties, or removal of prey from human‐laid snares (Brand et al., 2014).

In cases where prey body weight was not reported, we used the low end of adult male prey presented in faunal studies from the same area or referred to Kingdon et al. (2013). To account for infant, juvenile, and subadult prey, mean adult female body weight was multiplied by 75% [following Jooste et al. (2013)]. Mean adult male chimpanzee body weight (41.2 kg, *n* = 43; obtained from Thompson & Wrangham, 2013) was used to compare chimpanzee body weight with prey body weight, and the protein requirements of chimpanzees. Note that we use adult male chimpanzee body weight in contrast to other prey preference studies because they do most of the hunting (Gilby et al., 2017).

Jacobs’ selectivity index (*D*; Jacobs, 1974) was used to determine chimpanzee prey preferences for each prey species at each site. This involved calculating the proportional abundance of each prey species at each site from the total number of prey (*p*) and the proportion of the kills that species comprised of all chimpanzee kills from the total number of kill records of the particular site (*r*). These variables were used in the equation: *D* = (*r* − *p*)/(*r* + *p* − 2*rp*). The resulting value *D* is a score ranging from −1 (maximum avoidance) to +1 (maximum preference). Jacobs’ index diminishes the bias of rarer species by accounting for species rarity in relation to the total prey population at a given site and considering the heterogeneity of the confidence intervals (Jacobs, 1974). This metric also takes into consideration some of the other techniques, such as the forage ratio and Ivlev's electivity index (Ivlev, 1961). Jacobs’ index addresses the overstated accuracies in results presented and is preferred in determining the prey preferences of large carnivores and modern human hunter‐gatherers (Bugir et al., 2020; Hayward et al., 2017). Where data were normally distributed, we used *t* tests on the Jacobs’ index (*D*) values against a mean of 0 to determine if each prey species was significantly avoided or preferred. Where data were not normally distributed, we used a binomial sign test.

We tested for preferred and accessible weight ranges using breakpoint(s) in segmented models. Segmented models identify the ideal and preferred weight range of a predator, as well as which prey species fit within that range. Depending on the number of breakpoints, the change in slope between any two points determines changes of preference (Clements et al., 2014). The Jacobs’ index values of species either side of the breakpoints were tested for significant difference using a *t* test.

Maximum‐likelihood statistics through generalized linear models (glm function) were used to identify the factors that affected chimpanzee hunting decisions. To determine which models were strongly supported, we used Akaike's information criterion (Akaike, 1973, 1974) and the sum of their weights. The sum of Akaike's weights clarified the relative importance of each variable (i.e., prey body weight, hunt method, chimpanzee group size, sex ratio of chimpanzee group) in driving the Jacobs’ index value for each species. We used R statistical software 1.42.1. (R Core Development Team, 2016) and the *MuMIn* (Barton, 2018), *ggplot2* (Wickham & Chang, 2016), *segmented* (Muggeo, 2015), and *tidyverse* packages (Wickham, 2017).

## 3 RESULTS

**FIGURE 1:**
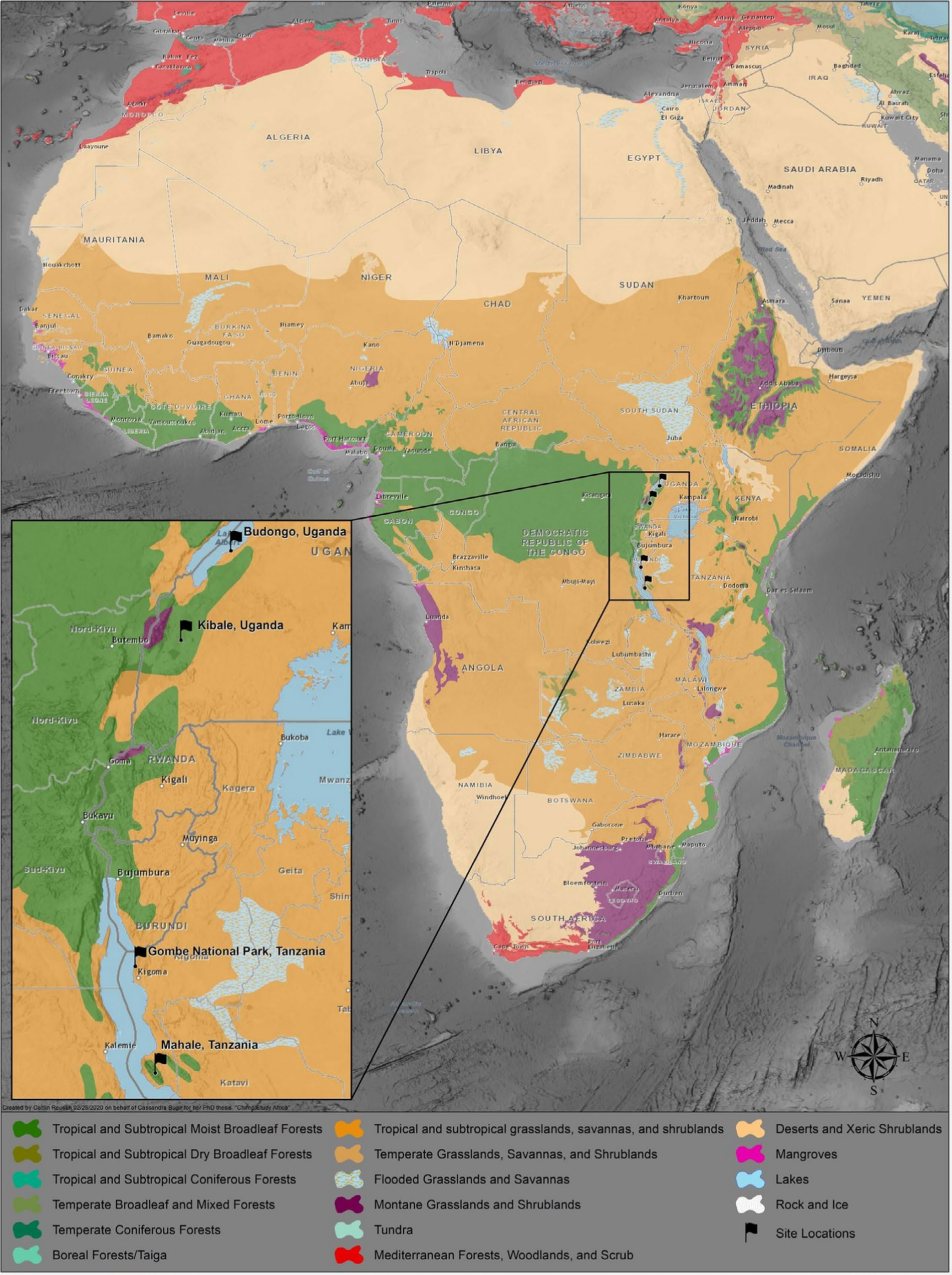
The four sites where data on chimpanzee predation were obtained for this study. Base map from Esri (2020)

We found 13 usable studies from two sites in Uganda and two sites in Tanzania (Table 1; Figure 1). These studies documented chimpanzee hunting from 1984 through 2017. Out of these 13 studies, we estimated Jacobs’ index values for 20 species that were hunted by chimpanzees across 76 times or places. Eleven of the 20 prey species had a sample size ≥3 kills reported. These 10 species were used for further analyses (Table 2).

**TABLE 1:** The four study sites used in this analysis and the species preyed upon by chimpanzees

Species recorded		Tanzania		Uganda	
		Mahale^a^	Gombe^b^	Kibale^c^	Budongo^d^
Baboon, olive	*Papio anubis*	x	x	x	x
Bushbuck (infant, juvenile)	*Tragelaphus sylvaticus*	x	x	x	—
Colobus, ashy red	*Piliocolobus tephrosceles*	x	x	x	—
Colobus, guereza	*Colobus guereza*	—	—	x	x
Duiker, blue	*Philantomba monticola*	x	—	x	x
Duiker, red	*Cephalophus callipygus*	—	—	x	x
Galago, Thomas's dwarf	*Galagoides thomasi*	—	—	x	—
Guineafowl	Numididae spp.	x	—	x	—
Bushpig (infant, juvenile)	*Potamochoerus larvatus*	x	x	x	—
Mangabey, gray‐cheeked	*Lophocebus albigena*	—	—	x	—
Monkey, gentle	*Cercopithecus mitis*	x	—	x	x
Monkey, L’hoest's	*Cercopithecus lhoesti*	—	—	x	—
Monkey, red‐tailed	*Cercopithecus ascanius*	x	x	x	x
Monkey, vervet	*Chlorocebus pygerythrus*	x	—	—	—
Rat, greater cane	*Thryonomys swinderianus*	x	—	—	—
Shrew, checkered elephant	*Rhynchocyon cirnei*	—	—	—	x
Squirrel	Sciuridae spp.	x	x	—	—
Warthog, common	*Phacochoerus africanus*	x	—	—	—

^a^ Hosaka et al. (2002), Newton‐Fisher et al. (2002), Takahata et al. (1984), Uehara (2003), Uehara & Ihobe (1998).

^b^ Gilby et al. (2017), Wrangham & Riss (1990).

^c^ Watts et al. (2012), Watts & Mitani (2015), Lwanga (2006), Lwanga et al. (2011), Teelen (2007).

^d^ Hobaiter et al. (2017), Newton‐Fisher et al. (2002).

**TABLE 2:** Preferred and avoided species that chimpanzees hunt

Common name	Scientific name	Body weight (kg)	*n*	Availability (%)	Kills (%)	*D*	*p*	Binomial (sign)	*t*
Baboon, olive	*Papio anubis*	24.4	5	0.12 ± 0.06	0.02 ± 0.01	−0.68 ± 0.45	.02		−3.85
Bushbuck (infant, juvenile)	*Tragelaphus sylvaticus*	10	6	0.06 ± 0.04	0.09 ± 0.03	0.51 ± 0.32	.09		2.03
Colobus, ashy red	*Piliocolobus tephrosceles*	7.6	4	0.23 ± 0.1	0.77 ± 0.04	0.59 ± 0.03	.01		3.5
Colobus, guereza	*Colobus guereza*	12.1	5	0.09 ± 0.04	0.22 ± 0.14	0.23 ± 0.31	.375	0.8	0.75
Duiker, blue	*Philantomba monticola*	8.9	8	0.3 ± 0.06	0.12 ± 0.04	−0.49 ± 0.16	.02		−2.99
Duiker, red	*Cephalophus callipygus*	11.5	4	0.22 ± 0.03	0.09 ± 0.07	−0.62 ± 0.16	.07		−2.68
Galago, Thomas's dwarf	*Galagoides thomasi*	0.06	1	0 ± 0	0.001 ± 0	—	1		
Guineafowl	Numididae spp.	0.7	3	0 ± 0	0.01 ± 0.001	—	.25		
Bushpig (infant, juvenile)	*Potamochoerus larvatus*	18	4	0.001 ± 0.001	0.05 ± 0.02	0.97 ± 0.17	.63	0.25	
Mangabey, gray‐cheeked	*Lophocebus albigena*	5.4	2	0.1 ± 0.09	0.01 ± 0.007	−0.59	.27		
Monkey, gentle	*Cercopithecus mitis*	5.8	7	0.07 ± 0.03	0.04 ± 0.02	−0.38 ± 0.10	.01		−3.25
Monkey, L’hoest's	*Cercopithecus lhoesti*	6	1	0.06 ± 0	0.001 ± 0	−0.97	1		
Monkey, red‐tailed	*Cercopithecus ascanius*	3.6	8	0.2 ± 0.06	0.04 ± 0.01	−0.55 ± 0.16	.01		−3.32
Monkey, vervet	*Chlorocebus pygerythrus*	5.9	2	0 ± 0	0.007 ± 0	—	.5		
Rat, greater cane	*Thryonomys swinderianus*	5.1	1	0 ± 0	0.003 ± 0	—	.25		
Sengi, checkered giant	*Rhynchocyon cirnei*	0.05	1	0 ± 0	0.005 ± 0	0	.25		
Squirrel	Sciuridae spp.	0.22	3	0.01 ± 0.01	0.01 ± 0.006	−0.15	1	0.33	
Warthog, common	*Phacochoerus africanus*	45	1	0.01 ± 0	0.04 ± 0	0.61	1		

Note

With Jacobs’ index (*D*), negative values indicate “avoided,” whereas positive values indicate “preferred.” Abundance (*p*) and mean kills (*r*) are proportions, including the standard error (± SE). “*n*” is the cumulative count of each species recorded from all of the sites.

**FIGURE 2:**
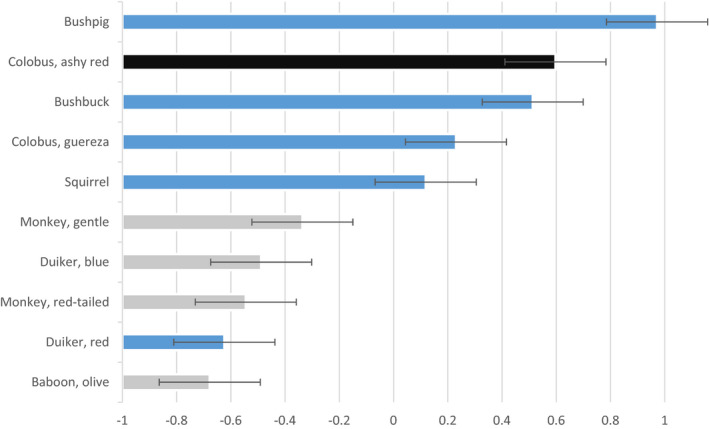
Chimpanzee prey preferences determined by mean Jacobs’ index values ±1 SE calculated from 13 studies at four sites. Significantly preferred prey, taken in excess of their abundance, are delineated by black bars. Gray bars denote significantly avoided prey which are less likely to be pursued irrespective of their abundance. Blue bars are prey that are taken or avoided according to their availability

The most significantly preferred prey of chimpanzees at three of the four study sites where they occur is the ashy red colobus monkey (*Piliocolobus tephrosceles*; Figure 2). Infant and juvenile bushbuck (*Tragelaphus scriptus*) and western guereza colobus monkey (*Colobus guereza occidentalis*) are taken in accordance with their availability (Table 2; Figure 2). It should be kept in mind that our study is based on snapshots of what chimpanzees preferred when the field study was conducted and, therefore, that these preferences will vary somewhat through time. We have found from other studies that prey preferences tend to be driven by evolutionary constraints rather than local conditions.

**FIGURE 3:**
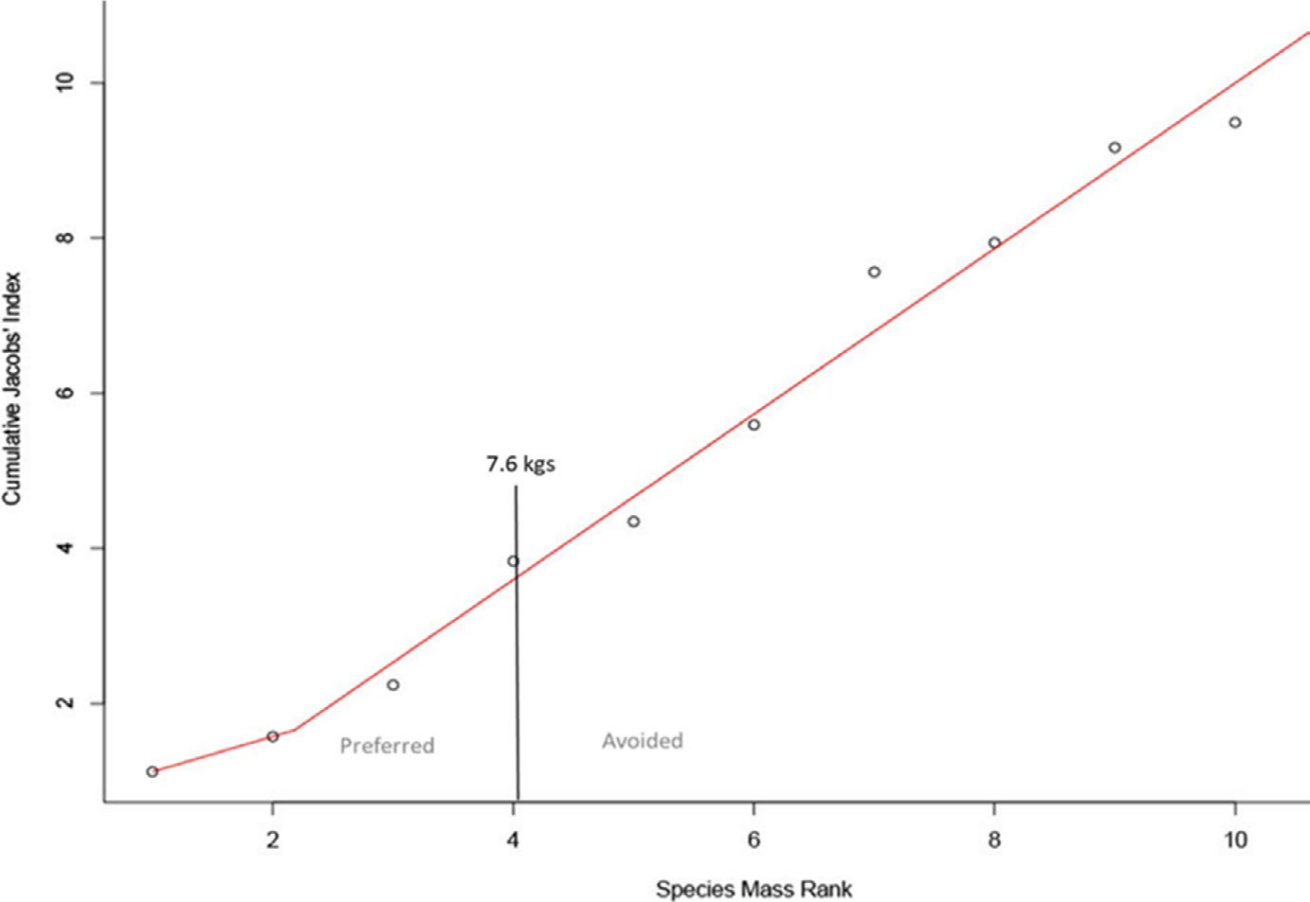
Segmented model plot of the cumulative Jacobs' index (CSJ) against body weight rank (SMR) of the 10 chimpanzee prey species with greater than three kill records (see Table 1). The breakpoint is at 7.6 kg, which corresponds to the body weight of ashy red colobus

Significantly avoided species are olive baboon (*Papio* *anubis*), blue duiker (*Philantomba monticola*), gentle monkey (*Cercopithecus mitis*), and red‐ tailed monkey (*Cercopithecus ascanius*; Table 2; Figure 2). The segmented model reveals only one breakpoint or point where the slope changes for prey preference (at 4.06). This corresponds to about the 7.6 kg threshold, as represented by ashy red colobus (Figure 3). Species below the 7.6 kg threshold are significantly preferred (*t* = −7.70, *df* = 5, *p* < .005), while those above are consumed in accordance with their availability in the prey community (*t* = −0.01, *df* = 6, *p* = .99). The ratio of ideal prey weight to adult male chimpanzee weight is 1:5.43 (18%).

**FIGURE 4:**
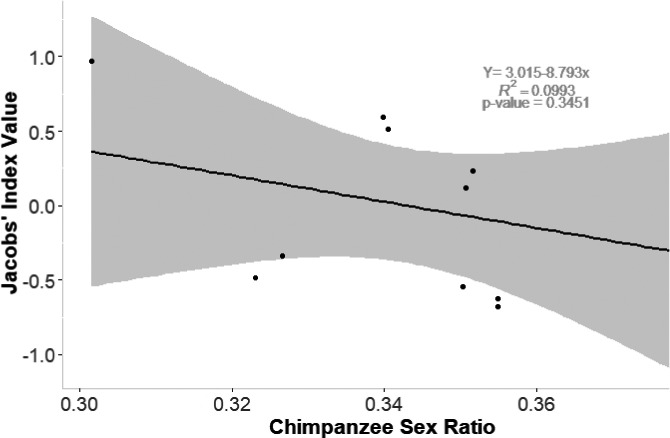
Sex ratio of chimpanzee groups studied, influencing Jacobs’ index for chimpanzee prey preferential selection. This was the most important variable in the AIC models, showing that the groups with sex ratios greater than 0.3 have fewer females to every male. A value of 0.3 implies that there are approximately two or more females to every male within one of the groups studied. This regression shows a steady decrease of preference with the more males in a group. This regression is, however, not statistically significant

The generalized linear model indicates that adult sex ratio of the entire chimpanzee group is the most important variable (sum of Akaike's weights *w* = 0.6) in developing prey preference (Table 3; Figure 4). The proportion of males to females in a group dictates how likely the chimpanzee group is to develop a preference and what prey are targeted. This sex ratio variable is twice as important as chimpanzee group size, prey body weight, or hunting method (Table 3).

**TABLE 3:** Model selection results from the generalized linear model for determining which factors are important in chimpanzee prey selection based on Akaike's information criterion corrected for small sample size (AICc)

Model	Intercept	Sex ratio of chimpanzee	Group size (chimpanzee)	Hunting method	Body weight (kg)	*df*	logLik	AICc	Δ	Weight
9	−0.845	1.943				3	−57.424	121.269	0	0.2
1	−0.185					2	−58.777	121.761	0.492	0.157
10	−0.898	2.263		+		5	−55.657	122.404	1.135	0.114
13	−0.725	2.143	−0.002			4	−56.871	122.457	1.188	0.111
5	−0.049		−0.001			3	−58.497	123.414	2.145	0.069
11	−0.86	1.951			0.001	4	−57.415	123.544	2.275	0.064
2	−0.142			+		4	−57.538	123.79	2.521	0.057
3	−0.189				0.001	3	−58.776	123.973	2.704	0.052
14	−0.83	2.339	−0.001	+		6	−55.526	124.607	3.337	0.038
15	−0.743	2.155	−0.002		0.002	5	−56.856	124.803	3.534	0.034
12	−0.91	2.269		+	0.001	6	−55.65	124.856	3.587	0.033
7	−0.055		−0.001		0.001	4	−58.495	125.703	4.434	0.022
6	−0.097		−0.0005	+		5	−57.508	126.107	4.838	0.018
4	−0.143			+	<0.001	5	−57.538	126.167	4.898	0.017
16	−0.844	2.349	−0.001	+	0.001	7	−55.516	127.145	5.876	0.011
8	−0.098		−0.0005	+	<0.001	6	−57.508	128.572	7.302	0.005
Null	0.844					4	−0.43	44.23	0	0.25
Importance:		0.6	0.31	0.29	0.24					
*N* containing models		8	8	8	8					

Note

“Weight” refers to the Akaike's weights or the likelihood of each model being the most supported in explaining the data. LogLik (log likelihood) refers to the parameters set within the model. Delta (∆) is the change from the AICc above, reflecting the contribution of additional parameters within the model. “Importance” (below the model numbers) refers to the sum of the Akaike's weights and is a relative measure of the support for each explanatory variable. Hunting method was the only categorical variable (either solitary or group hunting).

## 4 DISCUSSION

Like other predators, chimpanzees exhibit preferential predation (Boesch, 1994), but avoid prey that are too large to be worth capturing. The largest prey that chimpanzees prefer to hunt and consume is any species equal to or smaller than 7.6 kg, such as an ashy red colobus, where they are sympatric. Larger prey, such as adult olive baboons or large ungulates, are significantly avoided as they are too large to be safely captured by adult male chimpanzees (1:1.75 or 57%; Table 1; Harding, 1973). Yet, immature individuals of these species, which often fall within the preferred weight range, are hunted. In comparison, human hunter‐gatherers hunt prey weighing up to 276% of the weight of an adult female human (Bugir et al., 2020). Although meat is important food source for both species, it comprises a much larger part of the human diet [(approximately 60% among traditional human hunter‐gatherers (Butynski, 1982), including about 35% meat from mammals (Lee, 1968)] than in the chimpanzee diet (4%, although this varies among subspecies). Hunting for meat is an important evolutionary mechanism for driving larger brain sizes and innovation in hominids (Aiello & Wheeler, 1995). We conclude that chimpanzees are not apex predators of vertebrates in the way that modern humans, lions (*Panthera* *leo*), and tigers (*Panthera tigris*) are apex predators (Hayward et al., 2012; Hayward & Kerley, 2005).

According to the generalized linear model, the most important variable is the sex ratio of the group studied, with hunting more likely to yield broader prey preferences when the proportion of males is relatively high (Figure 3). Adult males are the primary hunters, although females sometimes join hunts or hunt with tools (Newton‐Fisher et al., 2007), much like human hunter‐gatherers (Hawkes & Bliege Bird, 2002). Hence, there are similarities between chimpanzees and humans, with both species possessing the intelligence, innovation with tool use, and skills to hunt and kill a vast array of prey species (Wood et al., 2017).

While that the drivers of chimpanzee hunting seem to be more social than dietary, the drivers of human hunter‐gatherer prey preferences appear to be optimal foraging upon species that can be captured effectively, minimizing energy expenditure while maximizing energy gain (Milner‐Gulland & Bennett, 2003). For modern human hunter‐gatherers, almost any prey within the range 2.5–535 kg (Bugir et al., 2020) is worth capturing to satisfy the optimal foraging imperatives of dietary protein requirements. This is because these people tend to live in “empty forests” (Redford, 1992) where prey populations are persistently over‐hunted.

Parallel to human hunter‐gatherers hunting and what they can find in “empty forests,” chimpanzees are exploiting red colobus at Ngogo, Kibale Forest, to the point where they may need to switch to new prey species (Watts & Mitani, 2015) or reduce their consumption of meat. Our results indicate that guereza and young bushbuck are taken in accordance with their availability. This suggests that they could be replacement prey should red colobus become over‐hunted at Ngogo. This suggestion may not necessarily translate to the other sites in this study nor to other *Pan* *troglodytes* subspecies.

Obtaining a baseline of chimpanzee preferences has the potential to aid in conservation management both of chimpanzees and their prey species. Understanding what prey is being targeted and what group is hunting provides the intrinsic information to bolster populations of prey and, consequently, chimpanzee populations. Protecting prey species allows us to obtain prey preference information and shed light on the factors driving the evolution of hunting and meat consumption in ancestral hominids.

## CONFLICT OF INTEREST

To the best of our knowledge, there is no conflict of interest.

## AUTHOR CONTRIBUTION

**Cassandra Bugir:** Data curation (lead); Formal analysis (lead); Writing‐original draft (lead); Writing‐review & editing (equal). **Thomas M** **Butynski:** Resources (equal); Validation (equal); Writing‐review & editing (equal). **Matthew Hayward:** Conceptualization (lead); Methodology (lead); Project administration (equal); Supervision (equal); Validation (equal); Writing‐review & editing (equal).

## Data Availability

Full dataset for chimpanzee study is available at: https://doi.org/10.5061/dryad.hdr7sqvhc.

## References

[ece37633-bib-0001] Aiello, L. C. , & Wheeler, P. (1995). The expensive‐tissue hypothesis: The brain and the digestive system in human and primate evolution. Current Anthropology, 36(2), 199–221. 10.1086/204350

[ece37633-bib-0002] Akaike, H. (1973). Second International Symposium on Information Theory. Akadémiai Kiadó.

[ece37633-bib-0003] Akaike, H. (1974). A new look at the statistical model identification. IEEE Transactions on Automatic Control, 19(6), 716–723. 10.1109/TAC.1974.1100705

[ece37633-bib-0004] Barton, K. (2018). MuMIn: Multi‐Model Inference. R package version 1.42.1. Retrieved from https://CRAN.R‐project.org/package=MuMIn

[ece37633-bib-0005] Boesch, C. (1994). Chimpanzees‐red colobus monkeys: A predator‐prey system. Animal Behaviour, 47(5), 1135–1148. 10.1006/anbe.1994.1152

[ece37633-bib-0006] Boesch, C. (2002). Cooperative hunting roles among Taï chimpanzees. Human Nature, 13(1), 27–46. 10.1007/s12110-002-1013-6 26192594

[ece37633-bib-0007] Brand, C. , Eguma, R. , Zuberbühler, K. , & Hobaiter, C. (2014). First report of prey capture from human laid snare‐traps by wild chimpanzees. Primates, 55, 437–440. 10.1007/s10329-014-0419-1 24682899

[ece37633-bib-0008] Bugir, C. K. , Peres, C. A. , White, K. S. , Montgomery, R. A. , Griffin, A. S. , Rippon, P. , Clulow, J. , & Hayward, M. W. (2020). Prey preferences of modern human hunter‐ gatherers. Food Webs, 26, e00183.

[ece37633-bib-0009] Butynski, T. M. (1982). Vertebrate predation by primates: A review of hunting patterns and prey. Journal of Human Evolution, 11, 421–430. 10.1016/S0047-2484(82)80095-X

[ece37633-bib-0010] Clements, H. S. , Tambling, C. J. , Hayward, M. W. , & Kerley, G. I. (2014). An objective approach to determining the weight ranges of prey preferred by and accessible to the five large African carnivores. PLoS One, 9(7), e101054. 10.1371/journal.pone.0101054 24988433 PMC4079238

[ece37633-bib-0011] Esri, HERE, Garmin (c) . (2020). OpenStreetMap contributors, and the GIS user community. General Bathymetric Chart of the Oceans (GEBCO); NOAA National Centers for Environmental Information (NCEI).

[ece37633-bib-0012] Gilby, I. C. , Machanda, Z. P. , O'Malley, R. C. , Murray, C. M. , Lonsdorf, E. V. , Walker, K. , Mjungu, D. C. , Otali, E. , Muller, M. N. , Emery Thompson, M. , Pusey, A. E. , & Wrangham, R. W. (2017). Predation by female chimpanzees: Toward an understanding of sex differences in meat acquisition in the last common ancestor of *Pan* and *Homo* . Journal of Human Evolution, 110, 82–94. 10.1016/j.jhevol.2017.06.015 28778463 PMC5570454

[ece37633-bib-0013] Gilby, I. C. , & Wawrzyniak, D. (2018). Meat eating by wild chimpanzees (*Pan* *troglodytes* *schweinfurthii*): Effects of prey age on carcass consumption sequence. International Journal of Primatology, 39(1), 127–140. 10.1007/s10764-018-0019-9

[ece37633-bib-0014] Goodall, J. (1986). The chimpanzees of Gombe: Patterns of behaviour. Belknap Press.

[ece37633-bib-0015] Harding, R. S. (1973). Predation by a troop of olive baboons (*Papio* *anubis*). American Journal of Physical Anthropology, 38(2), 587–591. 10.1002/ajpa.1330380272 4734718

[ece37633-bib-0016] Hawkes, K. , & Bliege Bird, R. (2002). Showing off, handicap signaling, and the evolution of men's work. Evolutionary Anthropology: Issues, News, and Reviews, 11(2), 58–67. 10.1002/evan.20005

[ece37633-bib-0017] Hayward, M. W. (2006). Prey preferences of the spotted hyaena (*Crocuta crocuta*) and degree of dietary overlap with the lion (*Panthera* *leo*). Journal of Zoology, 270(4), 606–614. 10.1111/j.1469-7998.2006.00183.x

[ece37633-bib-0018] Hayward, M. W. , Henschel, P. , O'Brien, J. , Hofmeyr, M. , Balme, G. , & Kerley, G. I. H. (2006). Prey preferences of the leopard (*Panthera pardus*). Journal of Zoology, 270, 298–313. 10.1111/j.1469-7998.2006.00139.x

[ece37633-bib-0019] Hayward, M. , Jędrzejewski, W. , & Jedrzejewska, B. (2012). Prey preferences of the tiger *Panthera* *tigris* . Journal of Zoology, 286(3), 221–231. 10.1111/j.1469-7998.2011.00871.x

[ece37633-bib-0020] Hayward, M. W. , & Kerley, G. I. H. (2005). Prey preferences of the lion (*Panthera* *leo*). Journal of Zoology, 267(3), 309. 10.1017/s0952836905007508

[ece37633-bib-0021] Hayward, M. W. , Porter, L. , Lanszki, J. , Kamler, J. F. , Beck, J. M. , Kerley, G. I. H. , Macdonald, D. W. , Montgomery, R. A. , Parker, D. M. , Scott, D. M. , O’Brien, J. , & Yarnell, R. W. (2017). Factors affecting the prey preferences of jackals (Canidae). Mammalian Biology, 85, 70–82. 10.1016/j.mambio.2017.02.005

[ece37633-bib-0022] Hobaiter, C. , Samuni, L. , Mullins, C. , Akankwasa, W. J. , & Zuberbühler, K. (2017). Variation in hunting behaviour in neighbouring chimpanzee communities in the Budongo forest, Uganda. PLoS One, 12(6), e0178065. 10.1371/journal.pone.0178065 28636646 PMC5479531

[ece37633-bib-0023] Hohmann, G. , Hublins, J. , & Richards, M. P. (Eds.). (2009). The diets of non‐human primates: Frugivory, food processing, and food sharing. In The evolution of hominin diets (pp. 1–14). Springer.

[ece37633-bib-0024] Hosaka, K. , Nishida, T. , Hamai, M. , Matsumoto‐Oda, A. , Uehara, S. , Galdikas, B. M. F. , Briggs, N. E. , Sheeran, L. K. , Shapiro, G. L. , & Goodall, J. (Eds.) (2002). Predation of mammals by the chimpanzees of the Mahale Mountains, Tanzania. In All apes great and small (pp. 107–130). Springer.

[ece37633-bib-0025] Ivlev, V. S. (1961). Experimental ecology of the feeding of fishes. Yale University Press.

[ece37633-bib-0026] Jacobs, J. (1974). Quantitative measurement of food selection. Oecologia, 14(4), 413–417. 10.1007/BF00384581 28308662

[ece37633-bib-0027] Jooste, E. , Hayward, M. W. , Pitman, R. T. , & Swanepoel, L. H. (2013). Effect of prey mass and selection on predator carrying capacity estimates. European Journal of Wildlife Research, 59(4), 487–494. 10.1007/s10344-013-0696-9

[ece37633-bib-0028] Kingdon, J. , Happold, D. C. D. , Butynski, T. M. , Hoffman, M. , Happold, J. , & Kalina, J. (Eds.). (2013). Mammals of Africa (6 volumes). Bloomsbury.

[ece37633-bib-0030] Lee, R. B. (1968). What hunters do for a living, or, how to make out on scarce resources. In R. B. Lee , & I. DeVore (Eds.), Man the hunter. Aldine.

[ece37633-bib-0031] Lwanga, J. S. (2006). The influence of forest variation and possible effects of poaching on duiker abundance at Ngogo, Kibale National Park, Uganda. African Journal of Ecology, 44(2), 209–218. 10.1111/j.1365-2028.2006.00629.x

[ece37633-bib-0032] Lwanga, J. S. , Struhsaker, T. T. , Struhsaker, P. J. , Butynski, T. M. , & Mitani, J. C. (2011). Primate population dynamics over 32.9 years at Ngogo, Kibale National Park, Uganda. American Journal of Primatology, 73(10), 997–1011. 21557287 10.1002/ajp.20965

[ece37633-bib-0033] Milner‐Gulland, E. J. , & Bennett, E. L. (2003). Wild meat: The bigger picture. Trends in Ecology & Evolution, 18(7), 351–357. 10.1016/S0169-5347(03)00123-X

[ece37633-bib-0034] Mitani, J. C. , & Watts, D. P. (2001). Why do chimpanzees hunt and share meat? Animal Behaviour, 61(5), 915–924. 10.1006/anbe.2000.1681

[ece37633-bib-0035] Muggeo, V. (2015). Regression models with breakpoints/changepoints estimation. Version 0.5‐1.2. Retrieved from: https://cran.r‐project.org/web/packages/segmented/index.html

[ece37633-bib-0036] Newton‐Fisher, N. E. , Henke, W. , & Tattersall, I. (Eds.) (2007). Chimpanzee hunting behavior. In Handbook of paleoanthropology (pp. 1295–1320). Springer.

[ece37633-bib-0037] Newton‐Fisher, N. E. , Notman, H. , & Reynolds, V. (2002). Hunting of mammalian prey by Budongo Forest chimpanzees. Folia Primatologica, 73(5), 281–283. 10.1159/000067454 12566760

[ece37633-bib-0038] Pilbeam, D. R. , Lieberman, D. E. , Muller, M. N. , & Wrangham, R. W. (2017). Reconstructing the last common ancestor of chimpanzees and humans. In Chimpanzees and human evolution, pp. 22–141.Harvard University Press.

[ece37633-bib-0039] Pruetz, J. D. , Bertolani, P. , Ontl, K. B. , Lindshield, S. , Shelley, M. , & Wessling, E. G. (2015). New evidence on the tool‐assisted hunting exhibited by chimpanzees (Pan troglodytes verus) in a savannah habitat at Fongoli. Sénégal. Royal Society Open Science, 2(4), 140507. 26064638 10.1098/rsos.140507 PMC4448863

[ece37633-bib-0040] Pyke, G. H. (1984). Optimal foraging theory: A critical review. Annual Review of Ecology and Systematics, 15(1), 523–575. 10.1146/annurev.es.15.110184.002515

[ece37633-bib-0041] R Core Development Team . (2016). R: A language and environment for statistical computing.

[ece37633-bib-0042] Redford, K. H. (1992). The empty forest. BioScience, 42(6), 412–422. 10.2307/1311860

[ece37633-bib-0043] Stanford, C. B. (1995). Chimpanzee hunting behavior and human evolution. American Scientist, 83(3), 256–261.

[ece37633-bib-0044] Stanford, C. B. (1996). The hunting ecology of wild chimpanzees: Implications for the evolutionary ecology of Pliocene hominids. American Anthropologist, 98(1), 96–113. 10.1525/aa.1996.98.1.02a00090

[ece37633-bib-0045] Stanford, C. B. , Wallis, J. , Mpongo, E. , & Goodall, J. (1994). Hunting decisions in wild chimpanzees. Behaviour, 131(1), 1–18. 10.1163/156853994X00181

[ece37633-bib-0046] Takahata, Y. , Hasegawa, T. , & Nishida, T. (1984). Chimpanzee predation in the Mahale Mountains from August 1979 to May 1982. International Journal of Primatology, 5(3), 213–233. 10.1007/BF02735758

[ece37633-bib-0047] Teelen, S. (2007). Influence of chimpanzee predation on associations between red colobus and red‐tailed monkeys at Ngogo, Kibale National Park, Uganda. International Journal of Primatology, 28(3), 593–606. 10.1007/s10764-007-9140-x

[ece37633-bib-0048] Thompson, M. E. , & Wrangham, R. W. (2013). *Pan**troglodytes* robust chimpanzee. In T. M. Butynski , J. Kingdon , & J. Kalina (Eds.), Mammals of Africa. Volume II: Primates, Vol. II (pp. 55–64). Bloomsbury.

[ece37633-bib-0049] Tomkins, J. P. (2016). Analysis of 101 chimpanzee trace read data sets: Assessment of their overall similarity to human and possible contamination with human DNA. Answers Research Journal, 9, 294–298.

[ece37633-bib-0050] Uehara, S. (2003). Population densities of diurnal mammals sympatric with the chimpanzees of the Mahale Mountains, Tanzania: Comparison between the census data of 1996 and 2000. African Study Monographs, 24(3), 169–179.

[ece37633-bib-0051] Uehara, S. , & Ihobe, H. (1998). Distribution and abundance of diurnal mammals, especially monkeys, at Kasoje, Mahale Mountains, Tanzania. Anthropological Science, 106(4), 349–369. 10.1537/ase.106.349

[ece37633-bib-0052] Watts, D. P. , & Mitani, J. C. (2015). Hunting and prey switching by chimpanzees (*Pan* *troglodytes* *schweinfurthii*) at Ngogo. International Journal of Primatology, 36(4), 728–748. 10.1007/s10764-015-9851-3

[ece37633-bib-0053] Watts, D. P. , Potts, K. B. , Lwanga, J. S. , & Mitani, J. C. (2012). Diet of chimpanzees (*Pan* *troglodytes* *schweinfurthii*) at Ngogo, Kibale National Park, Uganda, 1. Diet composition and diversity. American Journal of Primatology, 74(2), 114–129. 22109938 10.1002/ajp.21016

[ece37633-bib-0054] Wickham, H. (2017). Tidyverse: easily install and load the 'Tidyverse'. R package version 1.2.1. R Core Team. https://tidyverse.tidyverse.org/

[ece37633-bib-0055] Wickham, H. , Chang, W. , & Wickham, M. H. (2016). Package ‘ggplot2’. Create Elegant Data Visualisations Using the Grammar of Graphics. Version, 2(1) 1‐189. Rstudio.

[ece37633-bib-0056] Wood, B. M. , Watts, D. P. , Mitani, J. C. , & Langergraber, K. E. (2017). Favorable ecological circumstances promote life expectancy in chimpanzees similar to that of human hunter‐gatherers. Journal of Human Evolution, 105, 41–56. 10.1016/j.jhevol.2017.01.003 28366199 PMC5526083

[ece37633-bib-0057] Wrangham, R. W. , & Riss, E. V. Z. B. (1990). Rates of predation on mammals by Gombe chimpanzees, 1972–1975. Primates, 31(2), 157–170. 10.1007/BF02380938

